# Genetic Determinants of Autoimmune Myocarditis: From Candidate Genes to GWAS Insights

**DOI:** 10.3390/genes17070834

**Published:** 2026-07-21

**Authors:** Humza Pirzadah, Zainab Ibrahim, Yaseen Pirzadah, Nabiha Yusuf

**Affiliations:** 1 Louisiana State University Health Sciences Center School of Medicine, New Orleans, LA 70112, USA; 2 Heersink School of Medicine, University of Alabama at Birmingham, Birmingham, AL 35294, USA; 3 Department of Biological Sciences, Louisiana State University, Baton Rouge, LA 70112, USA; 4 Department of Dermatology and Epidemiology, University of Alabama at Birmingham, Birmingham, AL 35294, USA

**Keywords:** autoimmune, myocarditis, genetics, GWAS

## Abstract

Autoimmune myocarditis is an inflammatory disease of the myocardium driven by immune dysregulation and genetic predisposition. Recent advances in genomics, including genome-wide association studies (GWAS), have revealed key loci and pathways involved in disease susceptibility. This review synthesizes current knowledge on genetic determinants, from classical candidate genes to emerging GWAS findings, and explores their clinical implications for risk stratification and precision medicine.

## 1. Introduction

Myocarditis is an inflammatory disease that affects up to 14 per 100,000 people every year, with a mortality rate of up to 7% [1]. Although viral infections are the most common cause of myocarditis, autoimmune myocarditis is a significant cause of inflammatory cardiomyopathy [1,2]. Autoimmune myocarditis is a clinically significant condition that can progress to dilated cardiomyopathy and heart failure. While environmental triggers such as viral infections initiate the disease, genetic factors strongly influence susceptibility and outcomes [1,2]. The autoimmune characteristics of myocarditis are substantiated by various evidence that meet the Rose–Witebsky criteria for organ-specific autoimmune diseases [1,2]. Evidence includes the presence of immune cell infiltrates in endomyocardial biopsy (EMB) samples, the abnormal expression of HLA class II and adhesion molecules, circulating autoantibodies that target cardiac-specific antigens in both affected individuals and their relatives, as well as the capacity to provoke myocarditis through immunization with specific cardiac antigens in animal models [1,2,3]. Most cases are initially triggered by a viral infection; however, dysregulated persistent autoimmune responses drive cardiac injury and can progress to dilated cardiomyopathy (DCM) [2,4]. Although viral infections remain the most common initiating trigger of autoimmune myocarditis, other, non-viral factors also contribute. These factors include vaccines, medications, environmental triggers, immune checkpoint inhibitor therapy, and systemic autoimmune diseases, all of which can trigger immune-mediated injury in genetically vulnerable populations [2,4,5,6]. Sex-related differences have also been recognized in myocarditis pathogenesis. Although autoimmune disease is more common in females, myocarditis is more common in males, having a male-to-female ratio of roughly 2:1–4:1. Research suggests that these differences are attributed to hormonal differences, with testosterone enhancing pro-inflammatory pathways that increase myocarditis and fibrosis and estrogen promoting immune regulation and anti-inflammatory responses. Altogether, these findings suggest that sex can modify immune response and genetic susceptibility and should be examined as part of studying the pathogenesis of autoimmune myocarditis [2,7]. The diversity in clinical manifestations and outcomes has consistently indicated that genetic factors of the host are pivotal in influencing susceptibility to disease severity and progression. This review consolidates existing knowledge regarding the genetic factors associated with autoimmune myocarditis, ranging from traditional candidate gene research to novel findings from genome-wide association studies, and examines their clinical significance for risk assessment and precision medicine. Understanding these genetic underpinnings is essential for developing targeted therapies and improving patient care.

## 2. Pathogenesis of Autoimmune Myocarditis

**Figure 1:**
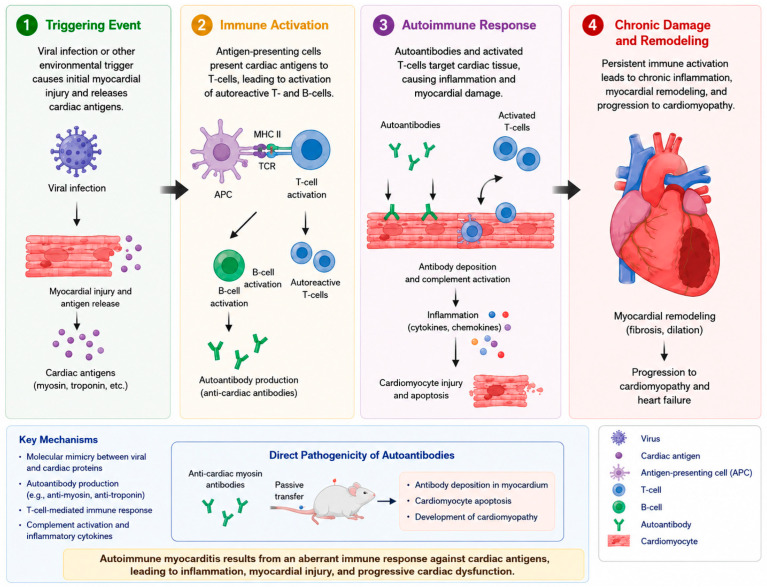
Pathogenesis of autoimmune myocarditis (created by AI).

Autoimmune myocarditis develops in phases. These phases shift the immune system from fighting infection to acting against self-antigens in the heart muscle. The various phases of pathogenesis of autoimmune myocarditis have been summarized in Figure 1.

Phase I: The pathogenesis of autoimmune myocarditis is multifactorial, involving both the innate and adaptive immune response [4,8]. Classically, immune-mediated myocardial injury involves a viral trigger from cardiotropic viruses, most notably coxsackievirus B3 (amongst many others) [1,2,8]. These cardiotropic viruses directly infiltrate cardiomyocytes, causing injury and releasing intracellular cardiac muscle proteins [2,8,9]. These proteins begin the initial inflammatory cascade by recruiting the innate immune system by activating type I interferon, natural killer cells, and nitric oxide pathways [2,4,8,9]. This initial damage to the tissue reveals cardiac antigens that were previously sequestered to the scrutiny of circulating immune surveillance [10].

Phase II: A critical mechanism that links viral infections to immune-mediated cellular destruction is molecular mimicry [11,12,13]. Viral coat proteins share homologous amino acid sequences with cardiac myocytes, leading to cross-reactive immune responses [10,11,12,13]. However, this is just one immunological mechanism; additional mechanisms encompass epitope spreading (the emergence of autoreactive responses to antigens liberated from injured tissue), bystander activation (the upregulation of costimulatory signals on antigen-presenting cells that activate pre-existing autoreactive clones), and the exposure of cryptic antigens subsequent to tissue damage [11,12,13].

Phase III: The dominant immune cell responsible for autoimmune myocarditis is α-myosin heavy chain (α-MyHC)-specific autoreactive T-cells [2]. These cells are present in all individuals since α-MyHC is not produced by medullary thymic epithelial cells (mTECs), leading to insufficient negative selection and the release of self-reactive clones into the peripheral circulation [2]. Under homeostatic conditions, these cells remain dormant as tissue-resident memory T-cells (TRMs) due to local regulatory signals and the expression of CD69 and PD-1 [2]. However, when these regulatory signals are disrupted due to pathologic insults such as viral infection, cardiac injury, systemic immune activation, or PD-1 checkpoint blockade, these TRMs become activated and release immune modulators (IFN-γ and TNF), leading to a cytotoxic response that injures cardiomyocytes and recruits additional inflammatory cells [2].

Phase IV: Cardiac-specific autoantibodies form in up to 60% of patients with inflammatory cardiomyopathy—these antibodies are also noted to be found in first-degree relatives, indicating a genetic predisposition to immune-mediated cardiomyopathy [2]. Primary antibody targets include α- and β-myosin heavy chain isoforms, β1-adrenergic receptors, muscarinic M2 receptors, cardiac troponin, and mitochondrial antigens [2]. These antibodies have shown direct pathogenicity and have also been shown to induce myocardial antibody deposition, cardiomyocyte apoptosis, and cardiomyopathy in recipient animals after passive transfer, showing that the genetic structure is a contributing factor to cardiomyopathy development [2,5].

Phase V: In genetically susceptible individuals, the breakdown of T-cell tolerance can lead to chronic autoantigen-driven inflammation that can progress to dilated cardiomyopathy and end-stage heart failure [8]. Recovery depends on the balance between regulatory and effector immune elements. CD4+ T-cell subsets exhibit unique temporal functions: Th1 (IFN-γ-producing) cells are predominant during the acute phase of autoimmune myocarditis, whereas Th17 responses are the primary contributors to the advancement of dilated cardiomyopathy (DCM) [4]. Chronic viral infections may worsen this phenomenon and have been linked to ongoing cardiomyopathy and prolonged production of anti-myosin antibodies, which lead to worsening cardiac injury [8,9]. Identification of the genes and protein involved in this process is essential for treatment strategy and prevention.

## 3. Genetic Architecture of Autoimmune Myocarditis

### 3.1. Major Histocompatibility Complex (MHC) Genes

The MHC region, which encodes the Human Leukocyte Antigen (HLA) molecule, is a strong genetic determinant of autoimmune myocarditis [2,5]. MHC polymorphisms affect the binding affinity of peptides derived from the self or pathogens to HLA molecules, which, in turn, influence immune tolerance and the variety of cardiac antigens that are presented to CD4+ T-lymphocytes [2,5]. Among HLA classes, class II has been strongly associated with autoimmune disease, specifically the HLA-DR4, HLA-DR12, and HLA-DR15 subclasses [2]. It has not only been associated with increased susceptibility to autoimmune myocarditis, but also a greater risk of progression to DCM [2,3]. A study conducted by Taylor et al. showed that HLA-DQ8 was involved in the predisposition to autoimmune myocarditis, as evidenced by a significant transgenic mouse model [14]. Expression of HLA-DQ8 alone in murine class-II-deficient mice led to the development of spontaneous autoimmune myocarditis, which was marked by lymphocytic infiltrates, the presence of circulating IgG autoantibodies targeting cardiac myosin heavy chain, and premature mortality due to heart failure despite the absence of an infectious trigger [14]. These findings indicate that the disease can be transferred through lymphocytes but not serum, suggesting that the condition is primarily mediated by T-cells rather than circulating antibodies. Additionally, specific HLA allele subtypes are associated with other cardiac pathogenicity. HLA-DPB*0901 and HLA-DRB1*1201 are enriched with hepatitis C DCM, thereby promoting persistent immune activation [15]. The allele HLA-DQB*0601 has been associated with cardiac sarcoidosis, potentially facilitating atypical antigen-driven granulomatous inflammation [16]. Certain HLA haplotypes have been linked to toxic myocarditis induced by clozapine as well as myocarditis associated with mRNA COVID-19 vaccines [5,17]. In this latter scenario, Aharon et al. (2024) discovered that DRB1*14:01 and DRB1*15:03, in conjunction with particular binding-groove motifs found in HLA-A and HLA-DR molecules, exhibited a significant correlation with myocarditis following vaccination [17]. This finding implies that immunogenetic signatures within peptide-binding grooves influence the presentation of vaccine-derived peptides to T-cells [5,17]. In the context of immune checkpoint inhibitor (ICI)-associated myocarditis, the HLA-A*01:01–B*08:01–C*07:01 haplotype has been associated with early onset ICI-induced myocarditis [18,19]. Furthermore, additional alleles such as HLA-DQB1*03:03, HLA-C*01:02, and HLA-B*52:01 have been observed more frequently among ICI-treated individuals who experience myocarditis [19]. Literature has shown that specific HLA gene variants may increase susceptibility to autoimmune myocarditis by promoting abnormal T-cell-mediated immune responses against cardiac tissue, thus indicating a strong genetic determinant in the development of autoimmune myocarditis (Table 1).

Recent evidence has also revealed anti-mitochondrial antibody (AMA)-positive myositis as an autoimmune disease entity correlated with a severe, distinct cardiac phenotype [20,21]. Cardiac manifestations include myocarditis, conduction abnormalities, cardiomyopathy, and ventricular arrythmias. Many patients with this disease require close surveillance and possible device therapy [20,21]. Although the exact basis of the genetic component of AMA-positive myositis remains unclear, its recognition broadens the spectrum of immune-mediated cardiac disease and emphasizes the role of autoantibody-defined phenotypes with genetic risk factors when assessing autoimmune myocarditis. As additional research clarifies the molecular mechanisms underlying AMA-associated cardiac disease, this subgroup may provide deeper insight into immune-mediated pathways involved in myocarditis and detect potential targets for clinical intervention.

### 3.2. Non-MHC Immunoregulatory Genes

Non-MHC genes have a greater impact on disease susceptibility compared to MHC genes in the development/severity of autoimmune cardiomyopathy [22]. Multiple non-MHC loci and immune checkpoint pathways have been identified through linkage analyses, knockout models, and studies involving pathway blockade, collectively outlining a complex polygenic framework that regulates T-cell activation thresholds, peripheral tolerance, and apoptotic control (Table 2). The immune checkpoint molecules PD-1 (programmed cell death protein 1) and CTLA-4 (cytotoxic T-lymphocyte-associated protein 4) are the most thoroughly studied non-MHC regulators of cardiac immune tolerance. The PD-1/PD-L1 pathway seems to play a cardioprotective role. Mice lacking Pdcd1 exhibited spontaneous and severe dilated cardiomyopathy, leading to early death [6]. This condition is marked by the deposition of IgG on cardiomyocytes, with antibodies subsequently identified as those that target cardiac troponin I [6]. PD-L1 is upregulated in cardiomyocytes, secondary to increased secretion of IFN-γ by infiltrating T-cells [23]. A PD-1 or PD-L1 deficiency leads to susceptibility to myocarditis due to a loss in cardio protection provided. In humans, more than 30 single nucleotide polymorphisms (SNPs) have been identified within the PDC1 gene, alongside several SNP regulatory proteins that alter binding sites for transcription factors [24,25]. These alterations have been associated with numerous autoimmune diseases, such as systemic lupus erythematosus, rheumatoid arthritis, multiple sclerosis, and Grave’s disease [24,25]. However, no direct association with isolated cardiomyopathy has been identified, and the cross-disease autoimmune association makes this a high-priority candidate locus for future investigation.

CTLA4 is another immune checkpoint inhibitor that has shown to be linked to autoimmune cardiomyopathies. CTLA-4 mainly restricts T-cell activation in lymphoid tissues during the priming phase by competing with CD28 for B7 ligands on antigen-presenting cells (APCs) [26]. In contrast, PD-1 reduces T-cell effector functions at tissue-specific locations, which is influenced by the constant expression of PD-L1 and PD-L2 [24,26]. Experimental models have shown that Ctla4 knockout mice exhibit fatal multiorgan autoimmunity within three weeks of age, characterized by autoimmune myocarditis that involves the infiltration of both CD4+ and CD8+ T-lymphocytes in the myocardium, regardless of genetic background [6,26]. This contrasts the sequence of PD-L knockout/deficiencies which took months to progress to organ dysfunction compared to weeks in the CTLA-4 knockouts [26]. In humans, CTLA-4 polymorphisms have been associated with many autoimmune diseases. The clinical prevalence of this association has been more noticeable with the increasing use of immune checkpoint inhibitors, leading to ICI-myocarditis in patients receiving anti-CTLA-4 (ipilimumab) therapy, particularly in combination with anti-PD-1 agents [12,23].

The ICOS (Inducible Costimulator) Pathway is a member of the CD28 family on activated T-cells that enhance the differentiation of effector T-cells, while also serving a dual purpose in maintaining/supporting the homeostasis and function of regulatory T-cells [27,28]. In experimental autoimmune myocarditis (EAM), the infiltrating cells expressed ICOS, especially CD4+ [29]. However, during the experiment, it was found that the timing of the ICOS/ICOSL pathway blockade determines the outcome. The blockade that occurred during the immune response phase (days 14–21) reduced the development of EAM, diminished the expression levels of IFN-γ, IL-4, IL-6, IL-10, IL-1β, and TNF-α, and inhibited the proliferation of T-cells [29]. Conversely, the blockade implemented during the antigen priming phase (days 0–14) unexpectedly worsened the disease, probably due to its detrimental effect on the formation of ICOS-dependent regulatory T-cells at the time of the initial antigen encounter [29]. This role has been further studied in ICOS-deficient mice models, which have shown that mice with the ICOS pathway blocked are more susceptible to EAM [30]. The protective function of ICOS is secondary to Treg activation: in knockouts, Tregs are unable to mature properly, leading to impaired ability to control Th-1-driven inflammation [30]. The ICOS/ICOSL pathway appears to play a complex immunoregulatory role in autoimmune myocarditis, with timing-dependent effects on inflammation through modulation of effector and regulatory T-cell responses.

CD45, which is encoded by the PTPRC gene, functions as a transmembrane protein tyrosine phosphatase that plays a crucial role in modulating Src family kinase signaling pathways within immune cells, thus establishing the activation threshold for T-cell receptors (TCRs) [31]. In humans, it has been reported that deletion or mutation of CD45 has been associated with dilated cardiomyopathy (DCM) and recurrent myocarditis [22,31]. There are two significant polymorphic variants that influence the expression of CD45 isoforms and susceptibility to diseases. The C77G polymorphism, which interferes with an exonic splicing, hinders the proper production of CD45 and has been linked to a higher occurrence of multiple sclerosis, autoimmune hepatitis, and systemic sclerosis [31,32,33]. The A138G polymorphism, which facilitates exon skipping, leads to an increased expression of the isoform CD45RO [31,33]. This variant has been associated with a protective effect against hepatitis B infection and autoimmune Graves’ thyroiditis [31,32,33]. Research involving transgenic mice has shown that modified combinations of CD45 isoforms, simulating the C77G and A138G human variants, lead to heightened severity of experimental autoimmune encephalomyelitis, with cells expressing CD45RO generating higher levels of TNF-α and IFN-γ [33]. However, limited reports are available on CD45 polymorphisms being directly related to development of myocarditis.

**Table 2:** Specific checkpoint genes and their clinically associated cardiomyopathies.

Checkpoint Gene	Knockout Phenotype	Mechanism of Cardiac Protection	Temporal Role	References
*PDCD1* (PD-1)	Spontaneous DCM with anti-troponin I antibodies (strain-dependent)	PD-L1 upregulated on cardiomyocytes by IFN-γ; limits effector T-cell function in tissue	Effector phase (tissue-specific)	[22,23]
*CTLA4* (CTLA-4)	Lethal multiorgan autoimmunity including myocarditis within 3 weeks	Competes with CD28 for B7 ligands on APCs; limits T-cell priming	Priming phase (lymphoid)	[6,23]
*ICOS* (ICOS)	Increased susceptibility to EAM due to impaired Treg activation and exaggerated Th1-driven inflammation	Supports Treg homeostasis and suppresses excessive effector T-cell responses	Dual role: immune response phase protective; priming phase regulatory	[28,30]
*PTPRC* (CD45)	Increased susceptibility to inflammatory and autoimmune disease due to dysregulated T-cell activation and enhanced pro-inflammatory cytokine production	Modulates Src family kinase signaling and establishes activation thresholds for T-cell receptor signaling	Regulates early T-cell activation and immune homeostasis	[31,32,33]

### 3.3. Structural Protein Genes

Cardiac structural proteins play a critical role in the inheritance of cardiomyopathy and inflammatory myocardial disease (Table 3). Of these structural proteins, the most commonly implicated genes are titin (*TTN*) and Desmoplakin (DSP) [1,34,35].

TTN is the largest protein in the human proteome and is responsible for structural integrity, passive elasticity, and force transmission in cardiomyocytes [33]. TTN truncating variants (TTNtvs) represent the most common genetic factor contributing to DCM, responsible for about 25% of familial cases and 18% of sporadic instances [34]. In the context of myocarditis, it was found that *TTN*tvs are enriched specifically in patients presenting with reduced LVEF [34,35]. This has been shown by meta-analytic data, which confirms that TTN variants are prevalent in myocarditis patients who exhibit acute heart failure, diminished LVEF, or ventricular arrhythmias [34,35,36,37]. TTN functions as both a mechanosensor and an immunomodulatory substrate [5,38]. Cytokine-induced changes in the post-translational modifications of titin can exacerbate the mechanical effects of TTNtvs and reveal previously compensated disease [5,38]. Systemic inflammation triggers the proteolytic cleavage of titin, resulting in fragments that may act as danger-associated molecular patterns (DAMPs), which further enhance innate immune responses and establish a feed-forward inflammatory cycle [33,35]. Truncated titin proteins are also incorporated into the sarcomere structure but lead to defects at the I/A junction and M-band, likely impairing mechanosensor functionality, leading to reduced LVEF [32,33,35]. *TTN*tvs create a genetically vulnerable myocardium that decompensates when exposed to inflammatory, hemodynamic, or toxic triggers, leading to the development of cardiomyopathies [34,39].

DSP links intermediate filaments to the desmosomal plaque, thereby enhancing cell-to-cell adhesion and mechanical strength [40]. Of all the genes associated with cardiomyopathy, DSP exhibits the strongest and most consistent correlation with myocarditis, as familial studies have consistently identified DSP in instances of recurrent myocarditis and in families experiencing both myocarditis and DCM or sudden cardiac death [37,38]. Unlike *TTN*tvs, truncating variants in DSP (DSPtvs) are associated with higher prevalence in patients who have preserved LVEF, but are still associated with ventricular arrhythmia [35]. DSP cardiomyopathy is now recognized as a distinct clinical entity characterized by left-dominant arrhythmogenic cardiomyopathy [41]. The mechanisms through which DSP variants induce inflammation are becoming increasingly well understood. Engineered heart tissues (EHTs) derived from DSPtv-hiPSCs and DSP^−^/^−^ cell lines exhibit baseline immune activation and cytokine release (through innate immune activation and NF-κB signaling) and exhibit heightened sensitivity to Toll-like receptor (TLR) stimulation, resulting in more significant contractile dysfunction compared to isogenic controls [42]. This phenotype can be ameliorated by colchicine or NF-κB inhibition [42]. On the contrary, cardiomyocytes lacking DSP accumulate cytosolic nuclear and mitochondrial self-DNA, which activates the cGAS-STING-IRF3/NF-κB DNA damage response pathway [43]. The genetic deletion of cGAS in DSP knockout mice has been shown to extend survival, enhance cardiac function, and reduce fibrosis [43]. Additionally, it was found by computational analysis that DSP is a crucial element of the MHC class I self-presentation complex. Truncating variants may disrupt the surface presentation of DSP peptides, leading to a loss of immune tolerance and an increase in autoimmune responses against cardiac antigens [44]. DSP mutations contribute to recurrent myocarditis and arrhythmogenic cardiomyopathy by promoting inflammation, immune dysregulation, and impaired structural integrity of cardiac tissue.

### 3.4. GWAS Insights and Polygenic Risk

**Figure 2:**
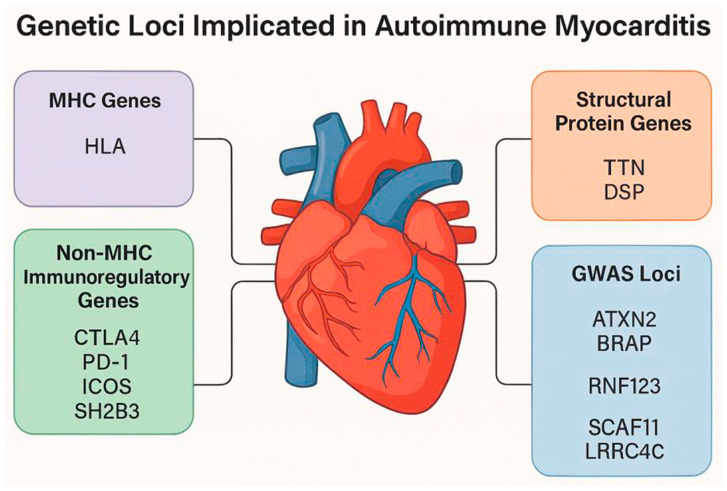
Genetic loci implicated in autoimmune myocarditis.

Genome-wide association (GWAS) studies have transformed our understanding of DCM and the genetic components that contribute to disease development and progression. The landscape of GWAS concerning myocarditis-related phenotypes has progressed rapidly. Approximately 70–80 significant genome-wide loci specifically uncovered for DCM have been identified (Figure 2) [45,46]. Furthermore, analyses focusing on tissue and pathway enrichment have validated the pivotal involvement of cardiomyocytes, the contractile apparatus, and immune-mediated pathways [45,46]. The landmark GWAS conducted by Meder et al. (2014), which examined more than 4100 cases of DCM alongside 7600 controls, pinpointed the first significant genome-wide susceptibility locus for idiopathic DCM located on chromosome 6p21 within the HLA region [47]. The most prominent signal was observed at rs9262636 (*p* = 4.90 × 10^−9^), located near the *HCG22* gene on chromosome 6p21 [47]. This SNP was recognized as an expression quantitative trait locus (eQTL) for various nearby genes that encode both MHC class I and class II heavy chain receptors, thereby establishing a direct functional connection between the genetic variant and immune-mediated pathogenesis [47]. This discovery was later validated in larger GWAS and multi-trait analyses, further supporting the role of genetically driven inflammatory processes as a factor in the pathogenesis of idiopathic DCM [45,46].

A significant discovery from cross-trait GWAS analyses is the recognition of common genetic loci that link autoimmune diseases with cardiovascular diseases, thereby offering a molecular foundation for the epidemiologically noted heightened cardiovascular risk associated with autoimmunity. The chromosome 12q24.12 locus serves as the most significant illustration of this pleiotropy, containing a group of genes such as SH2B3 (LNK), ATXN2, and BRAP, which are linked to various autoimmune and cardiovascular traits [48,49,50]. SH2B3 (LNK) is responsible for negatively regulating cytokine signaling and cell proliferation (endothelial and hematopoietic cells) through a lymphocytic adaptor protein [49,50,51]. The missense SNP rs3184504 (Pro262Trp) located in SH2B3 has been linked to many autoimmune diseases, coronary artery disease, myocardial infarction, and the LV end-diastolic internal dimension (through the associated SNP rs10774625 in ATXN2) [49,50,52]. Fine-mapping studies have pinpointed rs3184504 as the most probable causal variant at this locus, where SH2B3 (LNK) deficiency correlates with heightened platelet production and activation, as well as accelerated arterial thrombosis and atherosclerosis in hypercholesterolemic mice [53]. The BRAP gene located at the same locus was recognized in a Japanese GWAS as exhibiting the most significant association signal with coronary artery disease, with the association being particularly pronounced in cases of myocardial infarction [54].

GWAS focusing on adverse events related to COVID-19 vaccinations have started to uncover genetic factors that influence susceptibility to myocarditis. A GWAS involving 4545 Japanese participants identified 14 loci linked to vaccine-related adverse events, with the 6p21 (HLA) locus being associated with fever and muscle pain after mRNA vaccination [55]. Analysis of HLA allele associations indicated that HLA-DQA1*03:01 and HLA-A*11:01 were the most consistently linked to adverse effects [55]. Further candidate gene investigations have pointed to single nucleotide polymorphisms (SNPs) in immune checkpoint genes (CTLA4, CD28, PDCD1, TNFSF4) as contributors to vaccine side effects, with particular variants (rs3181096 and rs3181098 in CD28; rs733618 and rs3087243 in CTLA4; rs1234314 in TNFSF4) being associated with reactogenicity from both mRNA and adenoviral vector vaccines [56,57]. Moreover, genetic differences in KLRC2/NKG2C (which encodes an NK cell receptor) and TLR4 (rs4986790) have been linked to the risk and severity of adverse events following mRNA COVID-19 vaccination, highlighting the involvement of innate immune sensing pathways in vaccine reactogenicity [56,57]. Although preliminary analyses have reported specific GWAS signals near SCAF11 and LRRC4C in relation to vaccine-associated myocarditis, these results necessitate validation in larger, adequately powered cohorts before definitive conclusions can be drawn [55,56,57].

## 4. Insights from Experimental Models

Experimental models have been key in understanding the genetic architecture surrounding the development and progression of DCM. Two main model systems were developed: Coxsackievirus B3 (CVB3)-induced viral myocarditis and cardiac myosin-induced experimental autoimmune myocarditis (EAM). These models have been utilized to understand the susceptible loci, define the basis of genetic control, and have revealed mechanistic pathways with direct translational relevance to human disease [2,3,58].

The CVB3 infection model continues to be regarded as the gold standard for investigating viral myocarditis, as it accurately mirrors the biphasic progression seen in human cases: an acute phase mediated by the virus (days 10–14) marked by inflammatory infiltrates, myocyte necrosis, and left ventricular dysfunction, succeeded by a chronic immune-mediated phase (approximately day 30) that frequently leads to dilated cardiomyopathy with ongoing inflammation and fibrosis [2]. While the infectious virus is generally eliminated during the chronic phase, viral RNA and capsid proteins remain in the heart, spleen, and lymph nodes, sustaining inflammation through mechanisms that involve molecular mimicry and bystander activation [2]. Significantly, the pathogenic mechanisms vary even among susceptible strains that share the same MHC haplotype. In BALB/c mice (H-2d), cellular immunity, specifically cytolytic T-lymphocytes, serves as the main contributor to myocardial injury, as the depletion of complement does not affect the disease [59]. Conversely, in DBA/2 mice (also H-2d), heart-reactive autoantibodies are chiefly accountable, since the depletion of complement eliminates inflammation and necrosis [59]. This separation illustrates that the non-MHC genetic makeup of the host influences which effector arm of the immune system is responsible for cardiac damage, despite the MHC haplotype being the same [59]. Additionally, a quantitative trait locus (QTL) analysis conducted on segregating crosses between susceptible A/J and resistant B10.A mice has identified three distinct loci that regulate CVB3-induced myocarditis [59]. Vms1 located on chromosome 3 is associated with myocardial infiltration and sarcolemmal disruption in females [59,60]. Analysis of candidate genes has revealed Tnni3k (troponin I-interacting kinase), Fpgt (fucose-1-phosphate guanylyltransferase), and H28 as potential candidates that are developed within the Vms1 locus [59,60]. Notably, Tnni3k encodes a kinase specific to cardiac tissue that phosphorylates cardiac troponin I, thereby establishing a direct connection between a genetic susceptibility locus and the intrinsic vulnerability of cardiomyocytes [59,60]. Vms2 found on chromosome 1 and Vms3 located on chromosome 4 are associated with sarcolemmal disruption in males [60]. The sex-specific influences of Vms2 and Vms3 in contrast to Vms1 provide a genetic foundation for the well-established sex dimorphism in susceptibility to myocarditis and highlight the significance of sex as a biological variable in genetic research [60].

In the EAM model, disease is triggered by immunizing vulnerable strains with cardiac myosin or a myocarditogenic peptide sourced from α-myosin heavy chain (α-MyHC) emulsified in complete Freund’s adjuvant [2]. Inflammatory activity reaches its peak around day 21 and, in susceptible strains, advances to chronic-phase DCM between days 40 and 60 [2]. Histologically, the peak of the disease is characterized by a significant infiltration of leukocytes, which includes neutrophils, eosinophils, monocytes/macrophages, and many lymphocytes [2]. CD4+ T-cell-mediated responses are identified as the main contributors to myocardial injury [2]. The EAM model accurately reflects the histopathological features of giant cell myocarditis, which is marked by extensive myocardial damage and the presence of multinucleated giant cell infiltration [2]. Linkage analysis conducted in an A.SW mouse model has pinpointed two non-MHC loci that influence EAM susceptibility [61,62]. Eam1 located on murine chromosome 1 hS has shown that in congenic mice (B10.A-Eam1 congenic mice that possess the susceptible A.SW Eam1 locus on a resistant B10.S background), it is crucial in the progression of myocarditis [61,62]. Physiologically, Eam1 reduces lymphocyte apoptosis. It was shown that activation of caspase 3, 8, and 9 in lymph node cells following cyclophosphamide treatment and in CD4+ T-cells following immunization with myosin/CFA was significantly reduced in susceptible A.SW mice compared to resistant B10.S mice, with congenic mice displaying an intermediate phenotype [61,62]. Eam2 situated on murine chromosome 6 (separate from the MHC region) was identified through linkage analysis as a second factor that also regulates susceptibility [61]. Importantly, both Eam1 and Eam2 coincide with loci associated with autoimmune diabetes susceptibility as both influence apoptosis in thymocytes and peripheral T-cells [61]. These results confirm that the impaired apoptotic removal of autoreactive T-cells during both thymic and peripheral phases is a genetically influenced process common to various autoimmune disorders, including myocarditis [61,62].

## 5. Clinical Implications and Biomarkers

The integration of genetic testing, advanced imaging techniques, serological biomarkers, and novel targeted therapies is revolutionizing the clinical management of autoimmune myocarditis, shifting from empirical treatment to a precision medicine framework. The 2024 ACC Expert Consensus Decision Pathway now officially acknowledges genetic predisposition as a significant factor influencing the risk of myocarditis and advises that genetic testing ought to be included in the standard evaluation for new diagnoses [41]. The 2024 ACC Pathway advocates for genetic assessment for all individuals diagnosed with acute myocarditis, particularly emphasizing the need for prioritization in resource-constrained environments for those exhibiting recurrent myocarditis, possessing a family history of cardiomyopathy or sudden cardiac death, or displaying clinical “red flags” like non-sustained ventricular tachycardia during acute hospitalization and specific late gadolinium enhancement (LGE) patterns (ring-like or septal) observed on cardiac magnetic resonance (CMR) [41]. It is recommended that the genetic evaluation be conducted under the supervision of genetic specialists (clinical geneticists or genetic counselors) and should encompass the gathering of a three-generation family history along with molecular testing through targeted panel sequencing of established cardiomyopathy and inherited arrhythmia genes [41]. Given that genetic findings are unlikely to influence immediate clinical management, the evaluation should be carried out after the acute phase has resolved (post-hospitalization). A positive genetic test result (indicating a pathogenic or likely pathogenic variant) should initiate the following: cascade genetic testing for first-degree relatives, clinical evaluation for cardiomyopathy and arrhythmia in any relatives identified to carry the familial variant, and provision of guideline-directed medical therapy (GDMT) to previously undiagnosed relatives, which may enhance clinical outcomes [41]. Serum organ-specific anti-heart autoantibodies (AHAs) targeting myosin heavy chain and other autoantigens are recognized biomarkers for biopsy-confirmed autoimmune myocarditis, and present in as many as 60% of individuals diagnosed with inflammatory cardiomyopathy [2,41]. Functional investigations have shown that the passive transfer of anti-myosin antibodies results in myocardial antibody accumulation, cardiomyocyte apoptosis, and cardiomyopathy in recipient animals, thereby supporting a direct pathogenic involvement [2,3,12,41]. Nevertheless, validated cardiac autoantibody assays are not yet available for commercial use, which restricts their routine application in clinical settings [3,12,41]. However, there are many biomarkers that are under investigation.

Galectin-3 is known to be linked to cardiac remodeling and unfavorable prognosis in heart failure; its expression in human hearts correlates with myocarditis in Chagas cardiomyopathy, indicating a potential role in inflammation-induced fibrosis [41]. Soluble ST2 (sST2) is a member of the interleukin-1 receptor family that plays a role in cardiac remodeling and fibrosis [41]. Increased levels of sST2 have been associated with myocardial stress and negative outcomes across various cardiac conditions, and may offer insights into disease severity and prognosis in myocarditis [41]. Cellular immune-phenotype biomarkers are being explored, utilizing flow cytometry for a potential tool for myocarditis phenotyping; however, larger cohorts are pending [41]. MicroRNAs (miRNAs) are a growing yet still exploratory area where future prognostic/diagnostic understanding will evolve [41].

## 6. Future Directions

Future investigations into autoimmune myocarditis should focus on extensive multicenter genomic studies that incorporate GWAS, transcriptomics, proteomics, and epigenetic profiling to enhance the understanding of disease susceptibility and progression. Current findings indicate that autoimmune myocarditis is a heterogeneous and polygenic condition shaped by both immune-regulatory and structural cardiac genes; nevertheless, numerous proposed loci have yet to be adequately validated across various populations. Further research is required to elucidate the functional implications of identified variants, especially those related to immune checkpoint pathways, HLA subtypes, and structural proteins such as TTN and DSP. Progress in precision medicine may eventually facilitate personalized risk stratification, early identification of genetically predisposed patients, and targeted immunomodulatory treatments. Moreover, broadening research into circulating autoantibodies, microRNAs, and immune-cell phenotyping could enhance diagnostic precision and prognostic evaluation. Future translational studies that merge genetic insights with cardiac imaging and clinical outcomes will be essential for developing personalized prevention and treatment strategies.

## 7. Conclusions

Autoimmune myocarditis represents a multifaceted inflammatory cardiac condition influenced by genetic predisposition, immune system irregularities, and environmental factors. Recent advancements in candidate gene research, experimental autoimmune models, and genome-wide association studies have greatly improved our understanding of the molecular mechanisms that contribute to the onset and development of this disease. Significant insights from HLA-related immune regulation, non-MHC checkpoint pathways, and structural cardiac proteins like TTN and DSP underscore the complex nature of autoimmune myocardial damage. New genomic evidence further reinforces the idea that autoimmune myocarditis exists on a continuum that connects inflammation, hereditary cardiomyopathy, and immune-mediated cardiac impairment. As genomic technologies progress, the combination of genetic testing with biomarkers, sophisticated imaging techniques, and immunophenotyping is likely to enhance diagnostic accuracy, risk assessment, and targeted therapies. Ongoing translational research will be crucial for advancing precision medicine strategies and improving patient outcomes in those affected by autoimmune myocarditis.

## Figures and Tables

**Table 1:** Specific HLA subtypes and their clinically associated cardiomyopathies.

HLA Allele/Haplotype	Clinical Association	Key Mechanism	References
HLA-DR4	Myocarditis susceptibility; progression to DCM	Altered cardiac peptide presentation to CD4+ T-cells	[2,5]
HLA-DR12, HLA-DR15	Autoimmune myocarditis susceptibility	Enhanced autoreactive T-cell activation	[2]
HLA-DQ8 (DQA1\*0301*/*DQB1*\0302)	Spontaneous autoimmune myocarditis (transgenic model)	Sufficient alone to drive T-cell-mediated cardiac autoimmunity	[3,14]
HLA-DPB1*0901, HLA-DRB1*1201	Hepatitis C-associated DCM	Persistent immune activation via antigen presentation	[15]
HLA-DQB1*0601	Cardiac sarcoidosis	Abnormal antigen-driven granulomatous inflammation	[16]
DRB1*14:01, DRB1*15:03	mRNA COVID-19 vaccine myocarditis	Binding-groove motifs affecting vaccine peptide presentation	[17]
HLA-A*01:01–B*08:01–C*07:01	Early onset ICI myocarditis/myositis	Component of ancestral haplotype linked to multiple autoimmune diseases	[18]

(*) acts a separator in HLA nomenclature. It separates the gene name from the specific allele.

**Table 3:** Specific structural protein genes and their clinically associated cardiomyopathies.

Gene	Protein Function	Myocarditis Phenotype	Inflammatory Mechanism	References
*TTN*	Sarcomeric elasticity, mechanosensing	Reduced LVEF, heart failure	Titin fragments as DAMPs; cytokine-driven PTM alterations	[1,2,3]
*DSP*	Desmosomal cell adhesion	Preserved LVEF with arrhythmias; “hot phases”	NF-κB activation; cGAS-STING; altered MHC I self-presentation	[1,4,5,6]

## Data Availability

No new data were created or analyzed in this study. Data sharing is not applicable to this article.

## References

[1] Ammirati E., Moslehi J.J. (2023). Diagnosis and Treatment of Acute Myocarditis: A Review. JAMA.

[2] Jo W., Sun V., Čiháková D. (2026). Immune Mechanisms of Viral, Autoimmune, and Immune Checkpoint Inhibitor-Associated Myocarditis. Immunol. Rev..

[3] Law Y.M., Lal A.K., Chen S., Čiháková D., Cooper L.T., Deshpande S., Godown J., Grosse-Wortmann L., Robinson J.D., Towbin J.A. (2021). Diagnosis and Management of Myocarditis in Children: A Scientific Statement From the American Heart Association. Circulation.

[4] Won T., Song E.J., Kalinoski H.M., Moslehi J.J., Čiháková D. (2024). Autoimmune Myocarditis, Old Dogs and New Tricks. Circ. Res..

[5] Peretto G., Villatore A., Cooper L.T. (2026). Inflammation and Immunogenetics in Cardiomyopathies: From Molecular Mechanisms to Therapeutic Perspectives. Immunol. Rev..

[6] Lyon A.R., Yousaf N., Battisti N.M.L., Moslehi J., Larkin J. (2018). Immune checkpoint inhibitors and cardiovascular toxicity. Lancet Oncol..

[7] Fairweather D., Beatler D. (2023). Sex and gender differences in myocarditis and dilated cardiomyopathy: An update. Front. Cardiovasc. Med..

[8] Heymans S., Eriksson U., Lehtonen J., Cooper L.T. (2016). The Quest for New Approaches in Myocarditis and Inflammatory Cardiomyopathy. J. Am. Coll. Cardiol..

[9] Kociol R.D., Cooper L.T., Fang J.C., Moslehi J.J., Pang P.S., Sabe M.A., Shah R.V., Sims D.B., Thiene G., Vardeny O. (2020). Recognition and Initial Management of Fulminant Myocarditis: A Scientific Statement From the American Heart Association. Circulation.

[10] Zhao L., Fu Z. (2018). Roles of Host Immunity in Viral Myocarditis and Dilated Cardiomyopathy. J. Immunol. Res..

[11] Lasrado N., Reddy J. (2020). An overview of the immune mechanisms of viral myocarditis. Rev. Med. Virol..

[12] Sagar S., Liu P.P., Cooper L.T. (2012). Myocarditis. Lancet.

[13] Mone K., Reddy J. (2023). The knowns and unknowns of cardiac autoimmunity in viral myocarditis. Rev. Med. Virol..

[14] Taylor J.A., Havari E., McInerney M.F., Bronson R., Wucherpfennig K.W., Lipes M.A. (2004). A Spontaneous Model for Autoimmune Myocarditis Using the Human MHC Molecule HLA-DQ8. J. Immunol..

[15] Naruse T., Inoko H. (2000). HLA and hepatitis C virus positive cardiomyopathy. Jpn. J. Clin. Med..

[16] Naruse T., Matsuzawa Y., Ota M., Katsuyama Y., Matsumori A., Hara M., Nagai S., Morimoto S., Sasayama S., Inoko H. (2000). HLA-DQB1*0601 is primarily associated with the susceptibility to cardiac sarcoidosis. Tissue Antigens.

[17] Aharon A., Benedek G., Barhoum B., Parnasa E., Magadle N., Perzon O., Mevorach D. (2024). HLA binding-groove motifs are associated with myocarditis induction after Pfizer-BioNTech BNT162b2 vaccination. Eur. J. Clin. Investig..

[18] Müller-Jensen L., Flatz L., Hasan Ali O., Mohr R., Lachmann N., Mödl L., Endres M., Boehmerle W., Huehnchen P. (2025). HLA-A*01:01-B*08:01-C*07:01 is linked to early-onset immune checkpoint inhibitor-induced myositis and myocarditis. J. Immunother. Cancer.

[19] Lin X., Liu P., Jin M., Qi G. (2025). Clinical features and HLA typing of immune checkpoint inhibitor-associated myasthenia gravis, myocarditis and myositis. Front. Oncol..

[20] Mutoh T., Takahashi M., Hiroshi F. (2026). Anti-Mitochondrial Antibody-Positive Myositis: Analyses of 123 Adult-Onset Cases With Cardiac Evaluation. Int. J. Rheum. Dis..

[21] Mutoh T., Takahashi M. (2025). Anti-mitochondrial antibody-positive inflammatory myopathy with multiple arrhythmias resistant to high-dose glucocorticoids and intravenous cyclophosphamide: A case-based review. Clin. Rheumatol..

[22] Li H.S., Ligons D.L., Rose N.R. (2008). Genetic complexity of autoimmune myocarditis. Autoimmun. Rev..

[23] Grabie N., Lichtman A.H., Padera R. (2019). T cell checkpoint regulators in the heart. Cardiovasc. Res..

[24] Zamani M.R., Aslani S., Salmaninejad A., Javan M.R., Rezaei N. (2016). PD-1/PD-L and autoimmunity: A growing relationship. Cell. Immunol..

[25] Watanabe N., Nakajima H. (2012). Coinhibitory Molecules in Autoimmune Diseases. Clin. Dev. Immunol..

[26] Fife B.T., Bluestone J.A. (2008). Control of peripheral T-cell tolerance and autoimmunity via the CTLA-4 and PD-1 pathways. Immunol. Rev..

[27] Panneton V., Chang J., Witalis M., Li J., Suh W. (2019). Inducible T-cell co-stimulator: Signaling mechanisms in T follicular helper cells and beyond. Immunol. Rev..

[28] Chang J., Bouchard A., Bouklouch Y., Panneton V., Li J., Diamantopoulos N., Mohammaei S., Istomine R., Alvarez F., Piccirillo C.A. (2022). ICOS-Deficient Regulatory T Cells Can Prevent Spontaneous Autoimmunity but Are Impaired in Controlling Acute Inflammation. J. Immunol..

[29] Futamatsu H. (2003). Attenuation of experimental autoimmune myocarditis by blocking activated T cells through inducible costimulatory molecule pathway. Cardiovasc. Res..

[30] Dong C., Juedes A.E., Temann U.-A., Shresta S., Allison J.P., Ruddle N.H., Flavell R.A. (2001). ICOS co-stimulatory receptor is essential for T-cell activation and function. Nature.

[31] Hermiston M.L., Zikherman J., Zhu J.W. (2009). CD45, CD148, and Lyp/Pep: Critical phosphatases regulating Src family kinase signaling networks in immune cells. Immunol. Rev..

[32] Rong J., Yin J., Su Z. (2015). Natural antisense RNAs are involved in the regulation of CD45 expression in autoimmune diseases. Lupus.

[33] Dawes R., Petrova S., Liu Z., Wraith D., Beverley P.C.L., Tchilian E.Z. (2006). Combinations of CD45 Isoforms Are Crucial for Immune Function and Disease. J. Immunol..

[34] Monda E., Bakalakos A., Cannie D., O’Mahony C., Syrris P., Kaski J.P., Limongelli G., Elliott P.M. (2024). Prevalence of Pathogenic Variants in Cardiomyopathy-Associated Genes in Acute Myocarditis. JACC Heart Fail..

[35] Lota A.S., Hazebroek M.R., Theotokis P., Wassall R., Salmi S., Halliday B.P., Tayal U., Verdonschot J., Meena D., Owen R. (2022). Genetic Architecture of Acute Myocarditis and the Overlap With Inherited Cardiomyopathy. Circulation.

[36] Granzier H.L., Labeit S. (2025). Discovery of Titin and Its Role in Heart Function and Disease. Circ. Res..

[37] Burke M.A., Cook S.A., Seidman J.G., Seidman C.E. (2016). Clinical and Mechanistic Insights Into the Genetics of Cardiomyopathy. J. Am. Coll. Cardiol..

[38] Kellermayer D., Tordai H., Kiss B., Török G., Péter D.M., Sayour A.A., Pólos M., Hartyánszky I., Szilveszter B., Labeit S. (2024). Truncated titin is structurally integrated into the human dilated cardiomyopathic sarcomere. J. Clin. Investig..

[39] Kontorovich A.R. (2023). Approaches to Genetic Screening in Cardiomyopathies. JACC Heart Fail..

[40] Brandão M., Bariani R., Rigato I., Bauce B. (2023). Desmoplakin Cardiomyopathy: Comprehensive Review of an Increasingly Recognized Entity. J. Clin. Med..

[41] Drazner M.H., Bozkurt B., Cooper L.T., Aggarwal N.R., Basso C., Bhave N.M., Caforio A.L.P., Ferreira V.M., Heidecker B., Kontorovich A.R. (2025). 2024 ACC Expert Consensus Decision Pathway on Strategies and Criteria for the Diagnosis and Management of Myocarditis. J. Am. Coll. Cardiol..

[42] Selgrade D.F., Fullenkamp D.E., Chychula I.A., Li B., Dellefave-Castillo L., Dubash A.D., Ohiri J., Monroe T.O., Blancard M., Tomar G. (2024). Susceptibility to innate immune activation in genetically mediated myocarditis. J. Clin. Investig..

[43] Wang W., Cathcart B., Nguyen Q.D., Lao L.Q., Bryans A., Coleman S.E., Rouhi L., Gurha P., Marian A.J. (2025). Cardiomyocyte cytosolic nuclear self-DNA contributes to the pathogenesis of desmoplakin cardiomyopathy. JCI Insight.

[44] Mayfield J.J., Bogomolovas J., Abraham M.R., Sullivan K., Seo Y., Sheikh F., Scheinman M. (2023). Recurrent Myocarditis in Patients With Desmosomal Pathogenic Variants. JACC Clin. Electrophysiol..

[45] Zheng S.L., Henry A., Cannie D., Lee M., Miller D., McGurk K.A., Bond I., Xu X., Issa H., Francis C. (2024). Genome-wide association analysis provides insights into the molecular etiology of dilated cardiomyopathy. Nat. Genet..

[46] Jurgens S.J., Rämö J.T., Kramarenko D.R., Wijdeveld L.F.J.M., Haas J., Chaffin M.D., Garnier S., Gaziano L., Weng L.-C., Lipov A. (2024). Genome-wide association study reveals mechanisms underlying dilated cardiomyopathy and myocardial resilience. Nat. Genet..

[47] Meder B., Rühle F., Weis T., Homuth G., Keller A., Franke J., Peil B., Lorenzo Bermejo J., Frese K., Huge A. (2014). A genome-wide association study identifies 6p21 as novel risk locus for dilated cardiomyopathy. Eur. Heart J..

[48] Li X., Yan Z., Lan H., Wu Y., Chen S., Qiu G., Wu Y. (2025). Genetic Comorbidity of Psoriasis and Four Cardiovascular Diseases: Uncovering Shared Mechanisms and Potential Therapeutic Targets. Exp. Dermatol..

[49] Ochoa E., Iriondo M., Bielsa A., Ruiz-Irastorza G., Estonba A., Zubiaga A.M. (2013). Thrombotic Antiphospholipid Syndrome Shows Strong Haplotypic Association with SH2B3-ATXN2 Locus. PLoS ONE.

[50] Kullo I.J., Shameer K., Jouni H., Lesnick T.G., Pathak J., Chute C.G., De Andrade M. (2014). The ATXN2-SH2B3 locus is associated with peripheral arterial disease: An electronic medical record-based genome-wide association study. Front. Genet..

[51] Dichgans M., Malik R., König I.R., Rosand J., Clarke R., Gretarsdottir S., Thorleifsson G., Mitchell B.D., Assimes T.L., Levi C. (2014). Shared Genetic Susceptibility to Ischemic Stroke and Coronary Artery Disease: A Genome-Wide Analysis of Common Variants. Stroke.

[52] Wild P.S., Felix J.F., Schillert A., Teumer A., Chen M.-H., Leening M.J.G., Völker U., Großmann V., Brody J.A., Irvin M.R. (2017). Large-scale genome-wide analysis identifies genetic variants associated with cardiac structure and function. J. Clin. Investig..

[53] Dichgans M., Pulit S.L., Rosand J. (2019). Stroke genetics: Discovery, biology, and clinical applications. Lancet Neurol..

[54] Takeuchi F., Yokota M., Yamamoto K., Nakashima E., Katsuya T., Asano H., Isono M., Nabika T., Sugiyama T., Fujioka A. (2012). Genome-wide association study of coronary artery disease in the Japanese. Eur. J. Hum. Genet..

[55] Nogawa S., Kanamori H., Tokuda K., Kawafune K., Chijiiwa M., Saito K., Takahashi S. (2023). A web-based genome-wide association study reveals the susceptibility loci of common adverse events following COVID-19 vaccination in the Japanese population. Sci. Rep..

[56] Świerkot J., Tyczyńska K.M., Siemaszko J., Madej M., Sebastian A., Biały S., Morgiel E., Wielińska J., Sokolik R., Kujawa K. (2026). The association of selected genetic polymorphisms with adverse events following COVID-19 vaccination: A single-centre prospective observational cohort study. Virology.

[57] Chen D.-P., Wen Y.-H., Lin W.-T., Hsu F.-P. (2022). Association between the side effect induced by COVID-19 vaccines and the immune regulatory gene polymorphism. Front. Immunol..

[58] Wang Z., Mohamud Y., Luo H. (2026). Genetic factors contributing to viral myocarditis. Microbiol. Mol. Biol. Rev..

[59] Huber S.A., Lodge P.A. (1986). Coxsackievirus B-3 Myocarditis. Am. J. Pathol..

[60] Aly M., Wiltshire S., Chahrour G., Loredo Osti J.-C., Vidal S.M. (2007). Complex genetic control of host susceptibility to coxsackievirus B3-induced myocarditis. Genes. Immun..

[61] Guler M.L., Ligons D.L., Wang Y., Bianco M., Broman K.W., Rose N.R. (2005). Two Autoimmune Diabetes Loci Influencing T Cell Apoptosis Control Susceptibility to Experimental Autoimmune Myocarditis. J. Immunol..

[62] Ligons D.L., Guler M.L., Li H.S., Rose N.R. (2009). A locus on chromosome 1 promotes susceptibility of experimental autoimmune myocarditis and lymphocyte cell death. Clin. Immunol..

